# Similarities and differences between CLASS and ECERS-R estimates of educational environment quality

**DOI:** 10.3389/fpsyg.2023.1253154

**Published:** 2023-08-31

**Authors:** Daria Bukhalenkova, Olga Almazova, Margarita Aslanova

**Affiliations:** ^1^Department of Educational Psychology and Pedagogy, Faculty of Psychology, Lomonosov Moscow State University, Moscow, Russia; ^2^Department of Developmental Psychology, Faculty of Psychology, Lomonosov Moscow State University, Moscow, Russia

**Keywords:** preschool age, early care and education quality, learning environment, kindergarten quality, CLASS, ECERS-R

## Abstract

The conducted research was devoted to comparison of kindergartens’ educational environment quality evaluation via ECERS-R and CLASS methods. Both methods were applied in the same kindergarten groups. Therefore, in this study we attempted to find out if the educational environment quality assessments acquired via the two methods mentioned above would coincide. We analyzed the results from the cultural-historical psychology perspective. The educational environment quality assessment has been conducted in 83 Moscow kindergarten groups where study 5 to7 years old preschoolers. The correlation analysis results show that the ECERS-R method subscales are not related to the “Emotional support” CLASS domain, however, a significant correlation with the total ECERS-R score has been revealed. The “Classroom Organization” CLASS domain has the highest number of correlations to the ECERS-R subscales (4) as well as to the total ECERS-R score. The “Instructional Support” domain is connected only to the Parents and Staff subscale within the ECERS-R method. As a result of comparing groups with relatively low and high quality of the educational environment, that were identified based on the evaluation via the ECERS-R and CLASS methods, a good agreement between the results has been revealed. However, a fairly large number of groups with high CLASS scores have made it to the pool of average-low ECERS-R scores, which demonstrates a non-linear connection between the educational environment quality evaluations according to these two methods. Research allows to conclude that the ECERS-R and CLASS approaches complement each other well.

## 1. Introduction

As shown by numerous longitudinal studies the preschool education quality plays the key role in preschoolers’ cognitive and social development (Mashburn et al., 2008; Hamre et al., 2014; Hemdan and Marzouk, 2015) as well as their further academic success (Hamre and Pianta, 2003; Vandell et al., 2010; Welsh et al., 2010; Hall et al., 2013; Weiland and Yoshikawa, 2013; Sylva et al., 2014). In this regard, the assessment of preschool care and education quality is crucial (Schad and Arnold, 2019; Krivtsova, 2022). Over the past few decades, various instruments for measuring the educational environment quality in kindergarten groups have been developed around the world (ECERS, CLASS, STR, etc.). For countries that do not have their own instruments for educational environment quality evaluation it is important to identify the most useful and effective tools.

In this article, we will focus on two methods used widely all over the world, for the educational environment quality evaluation in preschool institutions – Early Childhood Environment Rating Scale – Revised (ECERS-R) (Harms et al., 2005) and Classroom Assessment Scoring System (CLASS) (Pre-K level) method (Pianta et al., 2008). The research goal was to compare the assessments of preschool education acquired using methods mentioned above. This aim becomes especially relevant as more and more researchers highlight the absence of the connection between the educational environment quality assessments and the results of the cognitive and social abilities development in preschoolers (Brunsek et al., 2017; Burchinal, 2017; McDoniel et al., 2022). This evokes a need to understand the educational environment evaluation methods in greater detail. It is also crucial not to make generalized conclusions about the efficacy of all methods used.

Kindergartens’ educational environment quality has been conceptualized in terms of structural and process quality (Vandell and Wolfe, 2000; Brunsek et al., 2017). Structural quality assessment focuses on aspects of the physical kindergarten environment, daily schedule and staff-child ratios. Process quality assessment focuses on interactions that occur within the kindergarten environment.

The ECERS-R method allows to assess the overall educational environment quality (both structural and process aspects), which includes information on object-material arrangement of kindergarten space, daily routine, and the nature of child’s interaction with the environment as well as social surroundings. The ECERS-R (Harms et al., 2016) consists of the following 7 subscales: (1) Space and Furnishing, (2) Personal Care Routines, (3) Language – Reasoning, (4) Activities, (5) Interaction, (6) Program Structure, (7) Parents and Staff. Each scale includes several (from 4 to 10) indicators. Therefore the ECERS indicators cover the whole spectrum of conditions, in which children find themselves in kindergarten (Shiyan and Vorobieva, 2015).

As opposed to the ECERS-R method, the CLASS approach does not assess the availability of various materials, physical environment or security. It focuses on student-teacher interactions, as well as on what the teacher does with the materials at their disposal, how effectively teacher uses them. Regardless of the age group under consideration, the CLASS highlights 3 main domains: (1) Emotional support, (2) Classroom organization, (3) Instructional support. Each domain includes several (from 3 to 4) dimensions, in its turn each dimension is evaluated on a 7-point scale (there are 10 dimensions in total) (Pianta et al., 2008).

The criteria given within the CLASS method are based on research that shows that the student-teacher interactions are the main mechanism of children’s development and education (Pianta et al., 2002; Mashburn and Pianta, 2006; Hamre and Pianta, 2007). This fits well with the ideas of Vygotsky (1984) on the leading role of an adult in children’s mental development. According to Vygotsky (1984), learning leads to development. In fact, learning is a specifically organized communication between a child and an adult. The teacher is a carrier of cultural norms and values, the teacher is an example that children seek to imitate. As Vygotsky wrote, “Human behavior is a product of development … of a social connections and relationships system, collective forms of behavior, and social cooperation” (Vygotsky, 2005, p. 865). Therefore, as part of the educational environment quality evaluation, the most significant is the study of the social situation of development from the standpoint of cultural-historical psychology. The social situation forming in kindergarten groups is what the CLASS method is mostly focused on.

Because the ECERS-R and CLASS methods evaluate different aspects of educational environment quality in kindergartens, the comparison of estimates obtained using these techniques in the same kindergarten groups is of interest in this study. We consider these methods since they are the most recognized by the world community as well as the most commonly used in the research worldwide (Perlman et al., 2016; Brunsek et al., 2017; McDoniel et al., 2022). In this study, we have taken an attempt to find out if the assessments of the quality of the educational environment acquired via these two methods would coincide. So, despite the differences in targeted parameters, the two methods could demonstrate identical assessments of the overall level of the educational environment. Alternatively, the scores for the two methods could be different, which would show that they measure different aspects of the educational environment quality. There is also a third option (the most probable one) – if the assessments would partially intersect (correlate) and partially differ, which would allow us to conclude that the two methods complement each other. This kind of research task rarely becomes the focus of scientists’ attention (Mashburn et al., 2008; McDoniel et al., 2022), as most researchers study just one of the methods mentioned above (Setodji et al., 2018), while comparing the tools for assessing the quality of the educational environment can help to understand how these methods work, how interchangeable they are, and which one of them will be more effective for the educational environment quality evaluation.

## 2. Materials and methods

### 2.1. Sample

The educational environment quality assessment has been conducted in 83 senior and preparatory Moscow kindergarten groups where study 5 to 7 years old preschoolers Senior (5–6 years old) and preparatory (6–7 years old) kindergarten groups are the last two stages of preschool education in Russia.

The study included municipal kindergartens in districts characterized by similar levels of infrastructure and designed to accommodate families with primarily medium income. All kindergartens used the same educational program called “From birth to school” (Veraksa et al., 2019).

In all kindergarten groups there were approximately the same number of children (about 30) and 1 or 2 preschool teachers (most often 2, but they work in shift). The number of teachers per group has varied from 1 to 3 people. The teachers’ average age was around 42 years old (М = 41.9; SD = 10.3 y.o.), and their experience of working as a teacher in preschool institutions was 13 years on average (М = 12.9; SD = 9.7). 72.1% of teachers have higher pedagogical education, 16.3% of teachers have graduated from pedagogical colleges, and 5.7% have higher education not related to pedagogy (engineering, law, economics).

In this study, CLASS and ECERS-R methods were used to assess the educational environment quality.

The CLASS method (Pre-K level) (Pianta et al., 2008) implies at least four 30 min group observation cycles (120 min. Per group in total) done by a trained expert. After each observation the expert sets scores on a 7-point Likert scale for each of the 10 method dimensions. At the same time, scores 1 and 2 conditionally refer to the low level, scores 3–5 characterize an average level, and 6–7 scores refer to a high level of interaction quality. As a result, the arithmetical mean of scores obtained from all observations in one group is acquired on each dimension, and based on the values obtained, scores are calculated according to a specific formula for the three main domains of the methodology (such as Emotional support, Classroom organization, Instructional support). The CLASS method (Hamre et al., 2014; Murray and Pianta, 2015) has been tested in about 50 senior and preparatory kindergarten groups in Russia (Bukhalenkova and Almazova, 2022) and has revealed a connection with executive functions development in preschoolers (Veraksa et al., 2020; Bukhalenkova et al., 2022a, 2022b).

The ECERS-R method (Harms et al., 2005) is a techynique for expert assessment done according to a specially designed evaluation sheet. Using this sheet, the expert evaluates 43 indicators of the kindergarten educational environment, organized in 7 subscales: “Space and furnishing,” “Personal care routines,” “Language – reasoning,” “Activities,” “Interaction,” “Program structure,” “Parents and staff” each of which is evaluated on a 7-point scale, with 1–2 point representing inadequate quality, 3–4 points – satisfactory, 5–6 points – good and 7 points representing excellent quality (Harms et al., 2016). According to the method, the total score is calculated as an arithmetic mean for all sub-scales. The research community has made great efforts to localize and adapt ECERS-R for Russia (Shiyan et al., 2016). The methodology was tested on a sample of 1,357 kindergarten groups in 76 subjects of the Russian Federation (Shiyan et al., 2016, 2021).

### 2.2. Procedure

Independent, specially trained and certified experts were assessing the quality of the educational environment according to CLASS and ECERS-R methods during one academic year.

The research was approved by the Ethics Committee of the Faculty of Psychology at Lomonosov Moscow State University (the approval No: 2023/18).

### 2.3. Data analysis

The following statistical methods and procedures were used in this research: descriptive statistics for general data analysis as well as correlation analysis (Spearman’s correlation coefficient) to identify relationships between estimates by CLASS and ECERS; cluster analysis (K-means method) and Kruskal–Wallis and Mann–Whitney test to identify groups with different levels of educational environment quality; cross tabulation and chi-square to identify relationships of educational environment levels according to the CLASS and ECERS methods.

## 3. Results

### 3.1. The results of educational environment quality assessment using CLASS and ECERS-R methods

Based on the educational environment quality evaluation obtained via the CLASS methodology (Table 1), we can conclude that the lowest scores in teachers’ interaction with children in Russian kindergartens are observed within the Instructional support domain. As low and average scores on all three dimensions included in this domain were obtained. For the other two domains (Emotional support and Classroom organization), medium or high scores were obtained.

**Table 1:** Descriptive statistics for the CLASS domains and dimensions.

	M	SD	Min	Max
Positive climate	5.1	1.08	2.3	7.0
Negative climate	1.6	0.79	1.0	5.5
Teacher sensitivity	5.0	1.32	2.3	7.0
Regards for students’ perspectives	4.6	1.14	2.3	7.0
Emotional support	5.3	0.83	2.8	6.9
Behavioral management	5.0	1.29	1.8	7.0
Productivity	5.0	1.06	2.3	7.0
Instructional learning formats	4.6	1.09	2.0	7.0
Classroom organization	4.9	0.99	2.5	7.0
Concept development	2.5	1.11	1.0	5.7
Quality of feedback	2.8	1.09	1.0	5.3
Language modeling	2.9	1.11	1.0	5.7
Instructional support	2.7	1.03	1.0	5.4

The results of the educational environment quality evaluation obtained via the ECERS-R methodology (Table 2), show that on all scales Russian kindergartens are at the low or average level. According to the ECERS-R method, among the groups observed, there is none that would have had good or excellent educational environment quality (5–7 points).

**Table 2:** Descriptive statistics for the ECERS-R subscales.

	M	SD	Min	Max
Space and furnishing	3.2	0.56	2.0	4.5
Personal care routines	3.4	0.85	1.8	5.2
Language – reasoning	3.1	0.63	1.3	4.3
Activities	2.7	0.52	1.8	3.9
Interaction	3.5	0.82	1.6	5.2
Program structure	3.3	0.74	2.0	5.7
Parents and staff	3.1	0.71	1.8	5.2
Total score	3.1	0.47	2.1	4.1

With the help of Kolmogorov–Smirnov criterion, verification of the normality of the distribution of scores according to scales and parameters of both methods was performed. Not all scales’ data is distributed normally, which points at the need to use nonparametric criteria in the statistical analysis of the results.

### 3.2. Comparison of results by CLASS and ECERS-R methods

To compare the results of the educational environment quality evaluation using CLASS and ECERS-R methods, Spearman correlation coefficient was used. Based on the correlation analysis results (see Table 3), the “Classroom organization” domain has the highest number of correlations to the ECERS-R methodology scales (4 scales out of 7 and total score). The “Instructional Support” domain is connected to one scale only – Parents and Staff – of the ECERS-R method. None of the ECERS-R scales scores (except the total score), were significantly connected to the scores on “Emotional Support” domain of the CLASS method.

**Table 3:** The correlations between ECERS-R and CLASS estimates.

ECERS-R subscale/CLASS domain	Emotional support	Classroom organization	Instructional support
Space and furnishing	0.10	0.18	0.19
Personal care routines	0.18	0.27*	0.17
Language – reasoning	0.16	0.10	0.18
Activities	0.17	0.26*	0.16
Interaction	0.17	0.19	0.11
Program structure	0.12	0.22*	0.10
Parents and staff	0.14	0.27*	0.30**
Total score	0.32*	0.34*	0.15

As the next step, we compared the groups with different levels of educational environment quality according to CLASS and ECERS-R methods. Cluster analysis was used to identify groups with significantly different levels of environmental quality since we divided the groups based not only on the total score, but also on the main parameters of each of the methods (domains and subscales).

Using cluster analysis (K-means method), kindergarten groups were divided into 2 clusters according to the estimates based on the three domains of the CLASS method (Emotional Support, Classroom Organization, Instructional Support). 47 kindergarten groups (average scores) have made it to the first cluster, and 36 (high scores) have made it to the second. Using the Mann–Whitney criterion, it was found that there are significant differences in the scores for all three domains from different clusters (*p* < 0.001), which allows us to talk about the resulting clusters as different levels.

Using cluster analysis (K-means method), kindergarten groups were divided into 3 clusters based on ECERS-R subscales scores. 30 groups made it to the first cluster (low scores), 33 groups formed the second (low-average scores) cluster, and 20 kindergarten groups made up the third (average scores) cluster. With the help of Kraskel–Wallis criterion, it was demonstrated that there are significant differences in the scores for all seven subscales (Space and Furnishing, Personal Care Routines, Language – Reasoning, Activities, Interaction, Program Structure and Parents and Staff) in all clusters (*p* < 0.001), which allows us to view the resulting clusters as different levels.

Using the *χ*^2^ criterion, we have found out that the educational environment quality levels by CLASS and ECERS-R are connected (*χ*^2^ = 7.276, *p* = 0.026, Cramer’s *V* = 0.296) (Table 4). According to the data obtained, the majority of the groups with low ECERS-R scores have also received average (the lowest) scores by CLASS (73.3%). At the same time, the majority of the groups that received the highest ECERS-R scores (corresponding to the average level), the also got high CLASS scores. However, a fairly large number of groups with high CLASS scores have made it to the pool of average-low ECERS-R scores, which demonstrates a non-linear connection between the assessments of educational environment quality according to these methods.

**Table 4:** Comparison of education environment quality levels according to the ECERS-R and CLASS methods.

		Quality of the educational environment by CLASS	
		Average	High
Quality of the educational environment by ECERS-R	Low	22 (73.3%)	8 (26.7%)
	Low-average	18 (54.5%)	15 (45.5%)
	Average	7 (35.0%)	13 (65.0%)

## 4. Discussion

The goal of this research was to compare the results of the educational environment quality evaluation in kindergarten groups measured using ECERS-R and CLASS methods.

It is important to note that as the result of ECERS-R assessment, none of the kindergarten groups have received a high total score (most scores were low or average), while the same groups have received average or high scores based on CLASS method. The data obtained by both methods within this study are consistent with the Mashburn’s and colleagues results on a sample of 671 kindergarten groups from 11 USA states (Mashburn et al., 2008). Rather low ECERS-R total scores were also obtained in many countries (for example, in Netherlands (De Kruif et al., 2009), Bahrein (Hadeed, 2013), Brazil (Mariano et al., 2018), Columbia (Betancur et al., 2021) etc.), that lead researchers to propose changes in the stop-coding system of ECERS-R indicators assessment that we support (Fujimoto et al., 2018).

From our point of view, the reason behind low quality of the educational environment of Russian kindergarten groups according to ECERS-R is primarily due to the discrepancy between the structural environment parameters, and not due to the interaction within the group itself: the lack of rest, relaxation and emotional comfort spaces; insufficient number and variety of toy blocks, books and materials for creativity and play (Remorenko et al., 2017). For the better understanding of obtained data it is important to view the arrangement of ECERS-R method’s scales and indicators in detail. In this method, separate indicators cannot be attributed to physical environment or interactions only: the scores within each indicator point to the quantity of conditions, as well as their quality. For example, if the object-material conditions (e.g., toy blocks, books, places for privacy, etc.) are available, the group may already count on getting 3 score points, however receiving higher scores (5–7 points) is only possible if the environment reaches a new level: not only the objects are present, but they are also available to children, and there are conditions provided for children’s independent and creative use of these objects (Shiyan and Vorobieva, 2015). Such arrangement of scales may be considered a distinctive feature of this method, but we also see a certain restriction to it: if there is a lack of some objects/conditions in the group, then the quality of interaction is not assessed whatsoever [a low score for the parameter is given straightaway, and the parameters related to high scores (often related to the quality of interaction) are not analyzed].

Whereas the CLASS methodology does not assess the diversity of the material-object environment, but only how the teacher uses the available materials to interact with children. In this regard, the rather high CLASS scores in the Emotional support and Classroom organization domains are due to the fact that teachers in Moscow kindergartens are well able to organize the effective routine and create a positive emotional climate in kindergarten groups using the means available (through verbal and non-verbal communication with children, attention to their opinions and assistance in solving emerging difficulties). One of the reasons for such results may be the education received by Russian teachers, based on the cultural-historical psychology, which focuses on the importance of the “child-social adult” interactions for children development. The adult poses as a bearer of an ideal form (signs) which child masters, improving their primary form in the process of interaction, mimicking the adult. Children learn cultural signs in the process of communication and begin to use them to control their inner mental processes – Vygotsky (1934) called this mechanism of assimilation of signs – internalization. Signs are not invented by children, but are acquired by them in communication with adults. Thus, the sign first appears on the outer plane, on the plane of communication, and then passes into the inner plane, the plane of consciousness (Vygotsky, 2005). Vygotsky was the first to deduce a specific mechanism of environmental influence, which actually changes the child’s psyche, leading to the emergence of higher mental functions (Lektorsky, 2023). Thus, it is through the communication of an adult and a child that the development of the latter occurs, which is reflected in the CLASS methodology. And the fact that the indicators for CLASS are generally higher than for ECERS just shows the foundation of the Russian educational system.

In the “From birth to school” educational program, which is widely used in Moscow kindergartens, authors emphasized that the physical environment itself does not guarantee the presence of a child’s initiative and its realization; it is rather guaranteed by an adult (Veraksa et al., 2019). The “From birth to school” program emphasizes the role of how exactly the teacher offers the child new knowledge: what type of orientation he/she uses to master children mental actions (Burmenskaya, 2022), what teaching resources for forming concepts he/she uses (Veraksa et al., 2022), how he/she supports independence and initiative in typical activities of a preschool child (e.g., playing, cognitive activities, etc.) (Karabanova, 2022; Veraksa, 2022) which is also evaluated in the Instructional Support domain of the CLASS, method. This similarity is natural, since the authors of both CLASS method as well as of the “From birth to school” program used the ideas of Vygotsky (1934). In addition, the main reason for parents’ dissatisfaction with kindergartens in Moscow was not the lack of material support, but the quality of interaction between a parent and a teacher, between the teacher and a child as well as between children (Podyanova and Polivanova, 2022). Thus, the CLASS method logic better complements the cultural-historical psychology ideas and the principles of preschool educational system in Russia.

The correlation analysis results show that the scales of the ECERS-R method are not related to the “Emotional support” domain of the CLASS method, however, a significant correlation with the total score by ECERS-R has been revealed. With this, the presence of emotional support is a pervasive parameter in ECERS scales, so it is impossible to give a high score by a number of criteria if there is no evidence of an emotionally warm treatment of the child (Shiyan and Vorobieva, 2015). Indeed, we have not received high scores based on the ECERS-R method, which may be the reason for the lack of the correlation with this domain. At the same time, such pervasive parameter may have shown itself in the presence of a significant correlation between the “Emotional support” CLASS domain and the ECERS-R total score. This result is also consistent with those obtained by Mashburn et al. (2008).

It is with the CLASS method’s “Classroom organization” domain with which the largest number of correlations of the ECERS-R method scales has been revealed. It demonstrates that both methods assess educational environment qualities that are connected to all processes in the group (learning, free play, eating, etc.) more or less in the same way.

Interestingly it was shown that, the “Instructional support” domain of the CLASS method is connected with the “Parents and Staff” ECERS-R scale only, while we would rather assume its link to “Language - Reasoning” and “Activities” scales, which, probably, may be explained by the peculiarities of scoring according to the ECERS-R method. The “Parents and Staff” scale results are not seen as completely reliable, since an expert makes his/her evaluations based on teacher’s answers alone, not on his/her own observations, which reduces the objectivity of the data obtained. Therefore, it was decided to exclude this parameter from the latest versions of the ECERS method (ECERS-3) (Harms et al., 2015) and we will study the revealed connection.

The obtained data on the ratio of different levels of environment according to ECERS-R and CLASS indicate a nonlinear relationship of scores of the two methods, which, as the analysis carried out previously (Bukhalenkova and Almazova, 2022), repeatedly confirms the significance of using both methods for the educational environment quality evaluation. With this in mind, we assume that this nonlinear correlation could be explained by the differences in which parameters of the educational environment are assessed by these methods. We suppose that the richness of the kindergartens’ physical environment may not be directly related to the quality of interactions between the teacher and children in the kindergarten group.

The limitations of the study include a small number of observed groups, which did not allow us to conduct several types of analysis. The research did not consider the important characteristics of teachers, such as their work experience, level of education, beliefs about children’s development (Hamre et al., 2012), and personality traits (Mazilov and Kostrigin, 2022; Nikitin and Lavrenyuk, 2022). Moreover, an analysis of the relationships between CLASS and ECERS-R scores with the results of children’s cognitive and emotional development considering their mono- or bilingual status will be crucial (Tvardovskaya et al., 2022), we plan to focus our attention on this in the future. Also we are going to compare the assessments of the quality of kindergartens’ educational environment with data regarding teachers (their work experience, level of education, and beliefs about child development).

## 5. Conclusion

The results of the study showed that the educational environment quality assessments acquired via the CLASS and ECERS-R methods partially intersect (correlate) and partially differ, which allowed us to conclude that the two methods complement each other. Also the conducted comparison of the educational environment quality evaluation obtained via the CLASS and ECERS-R methods showed that the ECERS-R method does not fully reflect the procedural quality of the environment, however from the cultural-historical psychology perspective, procedural quality is more important for children’s development than the structural quality. The CLASS methodology, which is aimed at assessing the procedural quality only, better agrees with the ideas of Vygotsky and complements the ECERS-R method well.

The usage of both methods in practical work with preschool educational institutions, as well as in scientific research, will allow us to take the largest number of educational environment characteristics that are significant for the preschoolers’ development into account, and get more objective data on the educational environment quality in kindergarten groups, due to the fact that these two methods focus on different aspects of the educational environment.

Analysis of the study results shows that these two methods do not replace each other. We hope that the study will help researchers who are thinking about choosing a method for the educational environment quality evaluation to make this choice taking into account the analysis of both methods from the standpoint of cultural-historical psychology.

## Data availability statement

The raw data supporting the conclusions of this article will be made available by the authors, without undue reservation.

## Ethics statement

The studies involving humans were approved by the Ethics Committee of the Faculty of Psychology at Lomonosov Moscow State University (the approval No: 2023/18). The studies were conducted in accordance with the local legislation and institutional requirements. Written informed consent for participation in this study was provided by the participants’ legal guardians/next of kin.

## Author contributions

DB and OA contributed to the presented idea (i.e., research questions) and the theoretical framework. MA and DB was involved in data collection. MA have prepared the data for the analysis. OA verified the analytical methods and conducted the analyses. DB wrote the manuscript considering critical feedback and input from OA. All authors contributed to the article and approved the submitted version.

## Funding

The study has been supported by Russian Science Foundation (RSCF), project no. 23-78-30005.

## Conflict of interest

The authors declare that the research was conducted in the absence of any commercial or financial relationships that could be construed as a potential conflict of interest.

## Publisher’s note

All claims expressed in this article are solely those of the authors and do not necessarily represent those of their affiliated organizations, or those of the publisher, the editors and the reviewers. Any product that may be evaluated in this article, or claim that may be made by its manufacturer, is not guaranteed or endorsed by the publisher.
